# ABO blood group distribution in migraine patients; the relationship of phenotypes with migraine subtypes, clinical features, and white matter lesions: a retrospective case-control study

**DOI:** 10.1186/s12883-026-04872-y

**Published:** 2026-04-02

**Authors:** Ferhat Kilicaslan, Erman Altunisik, Yasemin Ekmekyapar Firat, Kemal Ozan Lule

**Affiliations:** 1 Neurology Department, Patnos State Hospital , Agri, Türkiye; 2 Neurology Department, Gaziantep State Hospital , Gaziantep, Türkiye; 3 Faculty Of Medicine Neurology Department , Gaziantep Sanko University, Gaziantep, Türkiye; 4 Internal Medicine Department, Faculty of Medicine, Gaziantep University , Gaziantep, Türkiye

**Keywords:** Migraine, ABO blood group, White matter lesions, Magnetic resonance imaging, Rh factor

## Abstract

**Background:**

Migraine is a common and disabling neurological disorder with a complex and multifactorial pathophysiology. While genetic and vascular mechanisms have been extensively studied, the potential role of the ABO blood group system in migraine susceptibility and clinical characteristics remains unclear. This study aimed to evaluate the distribution of ABO and Rh blood groups in patients with migraine and to investigate their associations with migraine subtypes, clinical features, and white matter lesions (WMLs).

**Methods:**

This retrospective case–control study included 411 migraine patients aged 18–50 years and 4319 control individuals from the same geographical region. Migraine diagnosis and classification were established according to the International Classification of Headache Disorders, 3rd edition. ABO and Rh blood groups were determined using the gel centrifugation method. Migraine subtypes, clinical characteristics, and the presence of WMLs on cranial magnetic resonance imaging (MRI) were recorded. Group comparisons, univariate and multivariable logistic regression analyses, and a sensitivity analysis restricted to female participants were performed.

**Results:**

Blood group B was significantly less frequent in migraine patients compared with controls (13.4% vs. 18.6%, *p* = 0.009; significant after Bonferroni correction). In multivariable logistic regression adjusting for age and sex, blood group B remained independently associated with lower odds of migraine (adjusted OR = 0.660, 95% CI: 0.488–0.893; *p* = 0.007). A sensitivity analysis restricted to female participants yielded a consistent result (OR = 0.679, 95% CI: 0.499–0.924; *p* = 0.014). No significant differences were observed between migraine patients and controls with respect to A, O, and AB blood groups or Rh status. ABO and Rh blood group distributions were not significantly associated with migraine subtypes, the presence of WMLs, headache duration, or monthly headache frequency.

**Conclusions:**

Blood group B was underrepresented among migraine patients compared with the control population from the same geographical region. No associations were identified between ABO or Rh blood groups and migraine subtypes, clinical headache characteristics, or white matter lesions. These findings should be considered hypothesis-generating and require confirmation in larger, multicenter studies.

## Background

Migraine is a common and complex neurological disorder characterized by recurrent headache attacks and a substantial disease burden. Its global prevalence is approximately 12%, and it ranks as the second leading cause of neurological disability worldwide, surpassed only by stroke [1–3]. Migraine consists of overlapping phases, including the premonitory, aura, headache, and postdrome phases, all of which contribute to functional impairment and reduced quality of life [2].

Although migraine was long considered a benign condition without lasting consequences, accumulating evidence suggests an association with structural brain changes [4]. In particular, white matter lesions (WMLs), which reflect microvascular injury and are thought to arise from gliosis, demyelination, and axonal loss, have been reported more frequently in individuals with migraine. These lesions have been linked to adverse cognitive and physical outcomes, especially in later life, and migraine has been proposed as a potential risk factor for their development [5].

The ABO blood group system, the first human blood group system to be identified, was discovered in the early 1900s [6]. The ABO gene is located on chromosome 9q34.2 and encodes glycosyltransferases responsible for the synthesis of A and B antigens through modification of the H antigen [7]. Beyond its role in transfusion medicine, the ABO blood group system has been associated with susceptibility to various diseases, particularly infectious, autoimmune, cardiovascular, and inflammatory conditions [8].

Notably, ABO blood groups have been linked to thrombotic and vascular diseases, partly through their influence on circulating levels of coagulation factor VIII and von Willebrand factor [9]. In addition, genetic variants within the ABO locus, such as rs505922, have been associated with cardioembolic and large-vessel ischemic stroke, but not with small-vessel disease, suggesting a selective role in macrovascular pathology [10].

Despite these vascular and genetic links, the relationship between the ABO blood group system and migraine remains poorly defined, and data regarding its association with migraine subtypes, clinical characteristics, and WMLs are limited. Therefore, in this study, we analyzed the distribution of ABO and Rh blood groups in a cohort of 411 patients with migraine and compared these distributions with those of healthy (control) individuals from the same geographical region. We further investigated the associations between blood group distribution and migraine subtypes, clinical features, and the presence of WMLs.

## Methods

### Study design and participants

This retrospective case–control study included 411 patients aged 18–50 years who were diagnosed with migraine and admitted to Patnos State Hospital in 2024. The control group consisted of 4319 individuals from the same geographical region whose ABO and Rh blood groups had been determined during routine clinical evaluations. Individuals in the control group had no documented diagnosis of migraine. Sex and age information was available for both the migraine and control groups and was included in comparative analyses.

Migraine diagnosis and classification were established according to the International Classification of Headache Disorders, 3rd edition (ICHD-3).

### Blood group determination

ABO and Rh blood groups were determined using the gel centrifugation method (Odak Diagnostics, Turkey), in accordance with the manufacturer’s instructions. Venous blood samples were collected in EDTA-containing tubes, and blood group analyses were performed in the hospital laboratory under standardized conditions. The study protocol was approved by the Department of Support Services, Provincial Health Directorate, Governorship of Ağrı, Türkiye (Approval No: E-85724094-929-246168351; date: 11 June 2024). Due to the retrospective nature of the study, the requirement for informed consent was waived.

### Cranial magnetic resonance imaging (MRI) assessment

All migraine patients underwent cranial MRI as part of routine clinical evaluation. The presence of WMLs was determined based on T2-FLAIR sequences as documented in formal radiology reports.

### Statistical analysis

Statistical analyses were performed using the Statistical Package for the Social Sciences (SPSS), version 22.0 (IBM Corp., Armonk, NY, USA). The distribution of continuous variables was assessed using skewness and kurtosis values. Continuous variables were expressed as mean ± standard deviation (SD), and categorical variables were presented as frequencies and percentages [n (%)].

Comparisons between two independent groups were performed using the independent samples t-test for continuous variables and the chi-square test for categorical variables. Comparisons among more than two groups were conducted using one-way analysis of variance (ANOVA), as appropriate. In addition, multivariable logistic regression adjusting for age and sex was performed to evaluate the independent association between blood group B and migraine status. The Hosmer–Lemeshow goodness-of-fit test was used to assess model calibration. A sensitivity analysis restricted to female participants was also conducted. To account for multiple comparisons across four ABO blood groups, Bonferroni correction was applied (adjusted significance threshold: *p* < 0.0125). A *p*-value < 0.05 was considered statistically significant.

## Results

A total of 4730 individuals were included in the study, comprising 411 migraine patients and 4319 control subjects. The demographic characteristics of the control and migraine groups are summarized in Table 1. The mean age was 32.19 ± 8.54 years in the control group and 30.45 ± 8.20 years in the migraine group (*p* < 0.001). The control group included 3108 women (72.0%) and 1211 men (28.0%), whereas the migraine group included 382 women (92.9%) and 29 men (7.1%). Sex distribution differed significantly between groups (χ² = 88.16, *p* < 0.001).

**Table 1:** Demographic characteristics of the control and migraine groups

Variable	Control group (*n*:4319)	Migraine group (*n*:411)	*p* value
Age, years, mean ± SD	32.19 ± 8.54	30.45 ± 8.20	< 0.001
Female, n (%)	3108 (72.0)	382 (92.9)	< 0.001
Male, n (%)	1211 (28.0)	29 (7.1)	

Age was compared using the independent-samples t-test. Sex distribution was compared using Pearson’s chi-square test

*SD* standard deviation

The clinical and radiological characteristics of the migraine patients are summarized in Table 2. The mean duration of migraine attacks was 34.04 ± 30.5 h, and patients experienced an average of 14.13 ± 7.6 headache days per month. Migraine with aura was present in 6.1% of patients, and white matter lesions (WMLs) were detected on cranial MRI in 14.1% of the migraine cohort.

**Table 2:** Clinical and radiological characteristics of the migraine group

Variable	Migraine group (*n*:411)
Attack duration, hours, mean ± SD	34.04 ± 30.5
Headache days per month, mean ± SD	14.13 ± 7.6
Migraine with aura, n (%)	25 (6.1)
WMLs, n (%)	58 (14.1)

Continuous variables are presented as mean ± SD, and categorical variables as n (%)

*SD* standard deviation, *WML* white matter lesion

The distribution of ABO blood groups and Rh factor according to migraine characteristics is presented in Table 3. No statistically significant differences were observed in ABO blood group distribution between patients with and without aura (*p* > 0.05). Similarly, Rh factor distribution did not differ according to aura status (*p* > 0.05). The presence of WMLs was also not associated with ABO blood group distribution, with comparable proportions of A, B, O, and AB blood groups among patients with and without WMLs (*p* > 0.05). In addition, Rh positivity and negativity did not differ significantly according to WML status (*p* > 0.05).

**Table 3:** Distribution of ABO blood groups and Rh factor according to clinical characteristics in patients

Characteristics (*n*:411)	Blood groups *n* (%)					Rh groups *n* (%)		
	A	B	O	AB	*p*	Rh+	Rh-	*p*
Without aura	180 (46.6)	49 (12.7)	131 (33.9)	26 (6.7)	0.278	334 (86.5)	52 (13.5)	0.988
With aura	9 (36.0)	6 (24.0)	8 (32.0)	2 (8.0)		22 (88.0)	3 (12.0)	
WML +	24 (42.9)	12 (21.4)	17 (30.4)	3 (5.4)	0.250	48 (82.8)	10 (17.2)	0.299
WML –	165 (46.5)	43 (12.1)	122 (34.4)	25 (7.0)		308 (87.3)	45 (12.7)	

Comparisons were performed using Pearson’s chi-square test

*WML* white matter lesion, *Rh+* Rhesus positive, *Rh−* Rhesus negative

The association between ABO blood groups (A, B, O, and AB) and study groups (migraine patients vs. controls) was initially evaluated using a 4 × 2 Pearson chi-square test. A statistically significant difference in overall ABO blood group distribution was observed between the patient and control groups (χ² = 8.23, df = 3, *p* = 0.041); however, the effect size was small (Cramer’s V = 0.042). To identify the blood group primarily contributing to this difference, an explanatory post-hoc 2 × 2 chi-square analysis was performed. This analysis demonstrated that blood group B was significantly less frequent in the migraine group than in the control group (13.4% vs. 18.6%; χ² = 6.92, *p* = 0.009), whereas no significant differences were observed for the other blood groups. After applying Bonferroni correction for four comparisons (adjusted significance threshold: *p* < 0.0125), the difference for blood group B remained statistically significant (*p* = 0.009). Based on this finding, subsequent analyses focused on comparisons between blood group B and non-B blood groups (A, O, and AB) (Table 4). Because the overall ABO comparison suggested that the observed difference was primarily driven by blood group B, the subsequent B versus non-B analysis was performed as an explanatory follow-up analysis rather than a confirmatory etiologic model.

**Table 4:** Comparison of blood group B and Rh factor frequency between patient and control groups

Characteristics (*n*:4730)		Patient (*n*:411)	Control (*n*:4319)	*p*
ABO groups	**0**	141 (34.3)	1370 (31.7)	**0.041**
	**A**	187 (45.4)	1826 (42.3)	
	**B**	55 (13.4)	804 (18.6)	
	**AB**	28 (6.8)	319 (7.4)	
B Group and others	**0**,** A**,** AB**	356 (86.6)	3515 (81.4)	**0.009**
	**B**	55 (13.4)	804 (18.6)	
Rh Groups	**Rh-**	55 (13.4)	555 (12.9)	0.759
	**Rh+**	356 (86.6)	3764 (87.1)	

Comparisons were performed using Pearson’s chi-square test

Bold *p*-values indicate statistical significance (*p* < 0.05)

*Rh+* Rhesus positive, *Rh-* Rhesus negative

In univariate logistic regression analysis, blood group B was associated with a significantly lower likelihood of migraine (OR = 0.666, 95% CI: 0.494–0.898; *p* = 0.008) (Table 5, Model 1). To assess whether this association was independent of demographic differences between groups, multivariable logistic regression adjusting for age and sex was performed. After adjustment, the association between blood group B and migraine status remained statistically significant (adjusted OR = 0.660, 95% CI: 0.488–0.893; *p* = 0.007), while both younger age (OR = 0.976, 95% CI: 0.964–0.988; *p* < 0.001) and female sex (OR = 0.177 for males vs. females, 95% CI: 0.118–0.265; *p* < 0.001) were independently associated with migraine (Table 5, Model 2). The Hosmer–Lemeshow test indicated adequate model fit (χ² = 12.061, df = 8, *p* = 0.148). As a sensitivity analysis, univariate logistic regression restricted to female participants (*n* = 3,490) yielded a consistent result (OR = 0.679, 95% CI: 0.499–0.924; *p* = 0.014), further supporting that the observed association was not driven by sex imbalance between groups. Subgroup analyses stratified by Rh factor were conducted; however, no consistent or statistically robust associations were identified.

**Table 5:** Logistic regression analysis for the association between blood group B and migraine status

	B	SE	Wald	OR	95% CI	*p*
Model 1: Univariate						
Blood group B (vs. non-B)	−0.407	0.153	7.109	0.666	0.494–0.898	**0.008**
Model 2: Adjusted for age and sex						
Blood group B (vs. non-B)	−0.415	0.154	7.279	0.660	0.488–0.893	**0.007**
Age (per year)	−0.024	0.006	14.681	0.976	0.964–0.988	**< 0.001**
Sex (male vs. female)	−1.731	0.206	70.716	0.177	0.118–0.265	**< 0.001**
Sensitivity analysis: Females only						
Blood group B (vs. non-B)	−0.387	0.157	6.049	0.679	0.499–0.924	**0.014**

The dependent variable was migraine status (patient vs. control). Model 1: univariate logistic regression. Model 2: multivariable logistic regression adjusted for age (continuous) and sex (categorical). Sensitivity analysis: univariate logistic regression restricted to female participants (*n* = 3,490). Hosmer–Lemeshow goodness-of-fit test for Model 2: χ² = 12.061, df = 8, *p* = 0.148

Bold *p*-values indicate statistical significance (*p* < 0.05)

*OR* odds ratio, *CI* confidence interval, *SE* standard error

## Discussion

In this study, the association between ABO blood group distribution and migraine status was comprehensively evaluated. Although a statistically significant difference in overall ABO blood group distribution was observed between migraine patients and control subjects, the small effect size suggests that the clinical relevance of this association is limited. Importantly, the small effect size (Cramer’s V = 0.042) indicates that the observed group difference was very weak. In a sample of this magnitude, even trivial differences may reach statistical significance; therefore, these findings should not be interpreted as evidence of a clinically meaningful or protective association. Explanatory post-hoc analyses indicated that this overall difference was primarily driven by the lower frequency of blood group B among patients with migraine. Previous studies have investigated the relationship between ABO blood groups and various neuropsychiatric disorders. In patients with multiple sclerosis, blood group AB has been reported as a potential risk factor, whereas blood group O may have a protective role [11]. Another study demonstrated that psychiatric disorders occurred nearly three times more frequently in individuals with blood group AB compared with non-AB individuals [12]. In addition, a population-based study conducted in the United States reported higher mean depression scores among individuals with blood group O [13]. These findings suggest that ABO blood groups may be associated with certain neuropsychiatric conditions.

The relationship between psychiatric disorders and blood groups has been interpreted in the context of neurotransmitter and enzyme metabolism, with genetic susceptibility potentially mediated through enzymatic and molecular pathways [12]. Neurogenic inflammation, involving mediators such as histamine and calcitonin gene-related peptide (CGRP), is widely accepted as a central mechanism in migraine pathophysiology [14]. However, whether the mechanisms proposed for the association between ABO blood groups and psychiatric disorders are also relevant to migraine remains uncertain. At present, this possibility is hypothetical and is not supported by direct evidence.

The inverse association observed between blood group B and migraine status should not be interpreted as evidence for a specific vascular, inflammatory, or neuroimmune mechanism. However, because the observed association was modest in magnitude, it would be inappropriate to interpret the finding as evidence of a protective effect. Although the association persisted after adjustment for age and sex, the limited effect size and observational design preclude causal interpretation. Rather, the results indicate a limited statistical association that does not support pathophysiological inference from the current data. Subgroup analyses stratified by Rh factor did not demonstrate a consistent or robust pattern of association, and no evidence was found to support a modifying effect of Rh factor on the relationship between ABO blood groups and migraine status. These Rh-stratified analyses were exploratory in nature and should be interpreted with caution.

The established association between ABO blood group distributions and thrombotic vascular diseases has prompted extensive investigation in cerebrovascular disorders. In one study, blood group AB was found to be significantly more frequent among patients with ischemic stroke [15]. Similarly, a study conducted in the United States reported that individuals with blood group AB had an approximately twofold increased risk of ischemic stroke [9]. Another study demonstrated that non-O blood group phenotypes were associated with a higher risk of stroke compared with the O phenotype [16]. These findings support the notion that ABO blood groups may influence vascular disease risk, although their relevance to migraine—particularly in the absence of overt cerebrovascular pathology—remains uncertain. Non-O blood groups have been consistently associated with higher circulating levels of von Willebrand factor (vWF) and coagulation factor VIII, both of which contribute to a prothrombotic state [9, 16]. Endothelial dysfunction has been implicated in the pathophysiology of migraine, particularly in migraine with aura [17]. However, blood group B is a non-O phenotype and would therefore be expected to carry a higher—not lower—thrombotic risk, which does not straightforwardly explain its underrepresentation among migraine patients. This apparent paradox underscores the complexity of the observed association and indicates that it cannot be readily explained on the basis of coagulation-related mechanisms alone.

Notably, ABO blood group distribution did not differ between migraine patients with and without white matter lesions. Given that non-O blood groups are associated with elevated levels of vWF and factor VIII and, consequently, with increased thrombotic tendency, this negative finding does not support a clear association between ABO blood groups and WML status in our cohort. However, the small number of patients with WMLs limits the statistical power of this subgroup analysis, and the finding should be interpreted with caution.

The role of ABO blood groups in migraine-related biological processes is not well defined. To date, no definitive pathophysiological association between ABO blood groups and migraine has been established, and the available evidence remains limited and inconsistent. Importantly, there is no existing evidence to support a biologically meaningful association between blood group B and reduced migraine susceptibility. In the present study, although a statistically significant difference in overall ABO blood group distribution was observed between patients and controls, the small effect size indicates that the clinical implications of this finding are modest. The present findings are epidemiological in nature and do not establish a biological mechanism. Future studies incorporating measurement of vWF levels, endothelial function markers, and ABO genotyping are needed to explore potential pathophysiological links.

### Limitations

Several limitations of this study should be acknowledged. First, the retrospective design limits causal inference. Second, although multivariable logistic regression adjusting for age and sex was performed, data on other potential confounders—including hormonal status, oral contraceptive use, smoking, obesity, and vascular comorbidities—were not available for the control group. Residual confounding therefore cannot be excluded. Third, although genetic susceptibility is discussed, direct genetic analyses of the ABO locus were not performed, and the findings therefore cannot be directly linked to specific genetic variants. Fourth, the control group comprised individuals whose blood group was determined during routine clinical evaluations and may therefore not fully represent the general healthy population. Some individuals in the control group may have had undiagnosed migraine or other comorbidities that could influence the observed blood group distribution. Future studies should ideally employ population-based control groups with systematic exclusion of migraine through validated screening instruments. Fifth, the relatively small number of patients in certain subgroups, particularly those with aura and WMLs, limited the statistical power to detect potential associations. The negative findings in these subgroups should be regarded as inconclusive and warrant further investigation in larger samples. The high female predominance in the migraine group may reflect healthcare-seeking behavior in the study region and limits the generalizability of the findings to male patients. The assessment of WMLs was based on formal radiology reports rather than a standardized research protocol with predefined rating scales; inter-rater reliability was therefore not assessed. Finally, the study population was derived from a single geographical region, which may limit the generalizability of the results.

## Conclusions

In this study, we investigated the relationship between ABO blood group distribution and migraine, including migraine subtypes, clinical characteristics, and white matter lesions, within a single geographical region. We observed that blood group B was less frequent among migraine patients than among control individuals from the same population. While this finding suggests a possible association, its clinical relevance appears limited, and it should be interpreted as hypothesis-generating. Further studies incorporating multivariable models, genetic analyses, and prospective designs are required to clarify the functional role of ABO blood groups in migraine susceptibility and pathophysiology.

## Data Availability

The datasets generated and/or analyzed during the current study are not publicly available due to institutional data protection policies but are available from the corresponding author on reasonable request.

## Abbreviations

WML: White matter lesion
MRI: Magnetic resonance imaging
ICHD-3: International Classification of Headache Disorders, 3rd edition
EDTA: Ethylenediaminetetraacetic acid
vWF: Von Willebrand factor
CGRP: Calcitonin gene-related peptide
FLAIR: Fluid-attenuated inversion recovery
OR: Odds ratio
CI: Confidence interval
SE: Standard error
SD: Standard deviation
ANOVA: Analysis of variance
Rh: Rhesus

## References

[1] Pescador Ruschel MA, De Jesus O. Migraine headache. In: StatPearls. Treasure Island (FL): StatPearls Publishing; 2024. Available from: NCBI Bookshelf. Accessed 15 Oct 2025. 32809622

[2] Aguilar-Shea AL, Díaz-de-Terán J. Migraine review for general practice. Aten Primaria. 2022;54(2):102170. 34798397 10.1016/j.aprim.2021.102208 PMC8605054

[3] Schramm S, Börner C, Reichert M, Baum T, Zimmer C, Heinen F, et al. Functional magnetic resonance imaging in migraine: a systematic review. Cephalalgia. 2023;43(2):3331024221128278. 36751858 10.1177/03331024221128278

[4] Bai X, Wang W, Zhang X, Hu Z, Zhang X, Zhang Y, et al. Hyperperfusion of bilateral amygdala in patients with chronic migraine: an arterial spin-labeled magnetic resonance imaging study. J Headache Pain. 2023;24(1):1–10. 37848831 10.1186/s10194-023-01668-0 PMC10583377

[5] Bashir A, Lipton RB, Ashina S, Ashina M. Migraine and structural changes in the brain: a systematic review and meta-analysis. Neurology. 2013;81(14):1260–8. 23986301 10.1212/WNL.0b013e3182a6cb32 PMC3795609

[6] Lesky E. Viennese serological research about the year 1900: its contribution to the development of clinical medicine. Bull N Y Acad Med. 1973;49(2):100–11. 4567269 PMC1806929

[7] Yamamoto F, McNeill PD, Hakomori SI. Genomic organization of human histo-blood group ABO genes. Glycobiology. 1995;5(1):51–8. 7772867 10.1093/glycob/5.1.51

[8] Chen Z, Yang SH, Xu H, Li JJ. ABO blood group system and the coronary artery disease: an updated systematic review and meta-analysis. Sci Rep. 2016;6:23250. 26988722 10.1038/srep23250 PMC4796869

[9] Zakai NA, Judd SE, Alexander K, McClure LA, Kissela BM, Howard G, et al. ABO blood type and stroke risk: the Reasons for Geographic and Racial Differences in Stroke Study. J Thromb Haemost. 2014;12(4):564–70. 24444093 10.1111/jth.12507 PMC4913462

[10] Williams FMK, Carter AM, Hysi PG, Surdulescu G, Hodgkiss D, Soranzo N, et al. Ischemic stroke is associated with the ABO locus: the EuroCLOT study. Ann Neurol. 2013;73(1):16–31. 23381943 10.1002/ana.23838 PMC3582024

[11] Lopetegi I, Muñoz-Lopetegi A, Arruti M, Prada A, Urcelay S, Olascoaga J, et al. ABO blood group distributions in multiple sclerosis patients from the Basque Country: O – as a protective factor. Mult Scler J Exp Transl Clin. 2019;5(4):2055217319888954. 10.1177/2055217319888957 PMC6859684 31798940

[12] Pisk SV, Vuk T, Ivezić E, Jukić I, Bingulac-Popović J, Filipčić I. ABO blood groups and psychiatric disorders: a Croatian study. Blood Transfus. 2019;17(1):66–71. 29517969 10.2450/2018.0266-17 PMC6343596

[13] Singg S, Lewis JL. Depression and blood types. Psychol Rep. 2001;88(3):725–6. 11508010 10.2466/pr0.2001.88.3.725

[14] Yuan H, Silberstein SD. Histamine and migraine. Headache. 2018;58(1):184–93. 28862769 10.1111/head.13164

[15] Lotz RC, Welter C da, Ramos S, Ferreira SA, Cabral LE, de França NL. ABO blood group system and occurrence of ischemic stroke. Arq Neuropsiquiatr. 2021;79(12):1070–5. 34852069 10.1590/0004-282X-ANP-2020-0219

[16] Dentali F, Sironi AP, Ageno W, Crestani S, Franchini M. ABO blood groups and vascular disease: a systematic review and meta-analysis. J Thromb Haemost. 2012;10(7):1319–28.

[17] Paolucci M, Altamura C, Vernieri F. The role of endothelial dysfunction in the pathophysiology and cerebrovascular effects of migraine: a narrative review. J Clin Neurol. 2021;17(2):164–75. 33835736 10.3988/jcn.2021.17.2.164 PMC8053543

